# *Fusobacterium nucleatum* infection-induced neurodegeneration and abnormal gut microbiota composition in Alzheimer’s disease-like rats

**DOI:** 10.3389/fnins.2022.884543

**Published:** 2022-09-16

**Authors:** Caixia Yan, Qilin Diao, Yuxi Zhao, Cheng Zhang, Xiaoya He, Ruijie Huang, Yan Li

**Affiliations:** ^1^State Key Laboratory of Oral Diseases, West China Hospital of Stomatology, National Clinical Research Center for Oral Diseases, Sichuan University, Chengdu, China; ^2^Department of Pediatric Dentistry, State Key Laboratory of Oral Diseases, West China Hospital of Stomatology, National Clinical Research Center for Oral Diseases, Sichuan University, Chengdu, China

**Keywords:** periodontitis, Alzheimer’s disease, *Fusobacterium nucleatum*, Aβ, gut microbiota

## Abstract

**Objective:**

To explore whether *Fusobacterium nucleatum* could lead to behavioral and pathological changes in Alzheimer’s disease (AD)-like model rat and whether they could affect the gut microbiota.

**Methods:**

The cognitive ability and alveolar bone loss of Sprague-Dawley (SD) rats were tested by Morris water maze and Micro-CT, respectively. HE staining and immunohistochemistry were used to analyze the pathological changes and Aβ1–42 in brains. Western blot was applied to detect the expression of p-Tau 181 in the brain. Limulus amebocyte lysate assay and PCR were performed to determine serum LPS level and whether *F. nucleatum* accessed the brain, respectively. The gut microbiota was analyzed by the 16S rRNA gene sequence.

**Results:**

Oral infection with *F. nucleatum* could induce increased alveolar bone loss and learning impairment in AD-like rats. Additionally, *F. nucleatum* exposure increased the Aβ1–42 expression by about one-fourth (*P* < 0.05), p-Tau181 by about one-third (*P* < 0.05), and serum LPS (*P* < 0.05) in AD-like rats. Moreover, *F. nucleatum* could change the gut microflora composition in AD-like rats, accompanied by a significant increase in the abundance of *Streptococcus* and *Prevotella*.

**Conclusion:**

Oral infection with *F. nucleatum* could contribute to abnormalities in cognitive ability and pathological change in the brain of AD-like rats, which may be related to abnormal gut microbiota composition.

## Introduction

Alzheimer’s disease (AD) is a multifactorial neuro-degenerative disease, which affects cognitive function and memory, is characterized by amyloid β (Aβ, formed by activities of β and γ secretase) and neurofibrillary tangles (NFTs, composed of over-phosphorylated tau protein), but its pathogenesis is not yet clear (Blennow and Zetterberg, 2018; Weller and Budson, 2018). Most studies supported that inflammatory response plays an important role in the pathogenesis of AD by promoting Aβ deposition and leading to neuron loss and cognitive dysfunction (Long and Holtzman, 2019). Recently, more researchers were involved in the relationship between periodontitis and AD. Epidemiological studies had shown that people with periodontitis were at increased risk for AD, while people with AD were more prone to periodontitis, tooth loss, and mucosal lesions due to cognitive decline and impaired oral health (Chen C.K. et al., 2017).

Periodontitis is a chronic inflammatory disease caused by pathogenic bacteria in subgingival biofilm, and its pathogenesis is related to aberrant host immune response and destruction of periodontal tissues (Socransky and Haffajee, 2002; Sczepanik et al., 2020). Although periodontitis is not fatal, periodontal pathogens could travel through the systemic circulation to various organs, leading to the development of some life-threatening diseases. Studies had found that the presence and severity of periodontitis are associated with the development of systemic diseases, such as AD (Matsushita et al., 2020), and *Porphyromonas gingivalis* (*P. gingivalis*), a frequently studied periodontal pathogen, has been reported to increase the risk of AD, symptoms associated with AD were relieved after anti-*P. gingivalis*-toxin treatment (Dominy et al., 2019).

*Fusobacterium nucleatum* (*F. nucleatum*, *F.n*), another important periodontal pathogen, could mediate its interpolymerization with other bacteria and adhesion to various host cells (Fardini et al., 2011; Li et al., 2021). Current studies confirmed that *F. nucleatum* plays an important role in the occurrence and development of colorectal cancer and chemotherapeutic resistance, and could also be isolated from septicemia-related infections, pelvic inflammatory disease, abscesses of the brain and other organs (Han et al., 2003; Gregory et al., 2015; Brennan and Garrett, 2019). In line with these data, Sparks Stein and coworkers demonstrated that antibody levels to *F. nucleatum* and *P. intermedia*, at baseline, significantly increased as compared to the controls and correlated with a declined cognitive function in AD patients (Sparks Stein et al., 2012). In recent years, studies have found that gut microbiota might play an important role in neurological diseases. Animal studies using germ-free mice had shown that mice lacking the microbes have abnormal brain development, learning and memory deficits, and anxiety-like behaviors, suggesting that gut microbiota played a key role in early brain development and adult neurogenesis (Desbonnet et al., 2014; Grover and Kashyap, 2014). The gut–brain axis is a communication pathway between the center neuro system and the enteric nervous system (Kowalski and Mulak, 2019). It is suggested that AD patients have different gut microbiota from non-AD people, and this abnormal gut microbiota may be involved in the deposition of the brain Aβ (Seo et al., 2019). Therefore, further studies on the gut microbiota of AD patients can help us better understand the etiology of the disease. In our study, *F. nucleatum* was orally infected D-galactose/AlCl_3_ induced AD-like rats, and we found that *F. nucleatum* changed the gut microbiota and played a pathogenic role in AD.

## Materials and methods

### Animals

Thirteen 5-week-old Sprague-Dawley (SD) male rats (130–150 g) purchased from the Dashuo company were housed in plastic cages in a temperature-controlled (25°C) colony room at a 12/12 h light/dark cycle. Food and water were available *ad libitum*. All animal procedures in this study were approved by the Ethics Committee of West China Hospital of Sichuan University (WCHSIRB-D-2021-009) and conformed to the ARRIVE (Animal Research: Reporting of *in vivo* Experiments) guidelines for preclinical studies. All efforts were made to minimize the number of animals used.

### Alzheimer’s disease-like and periodontitis rat model

**FIGURE 1:**
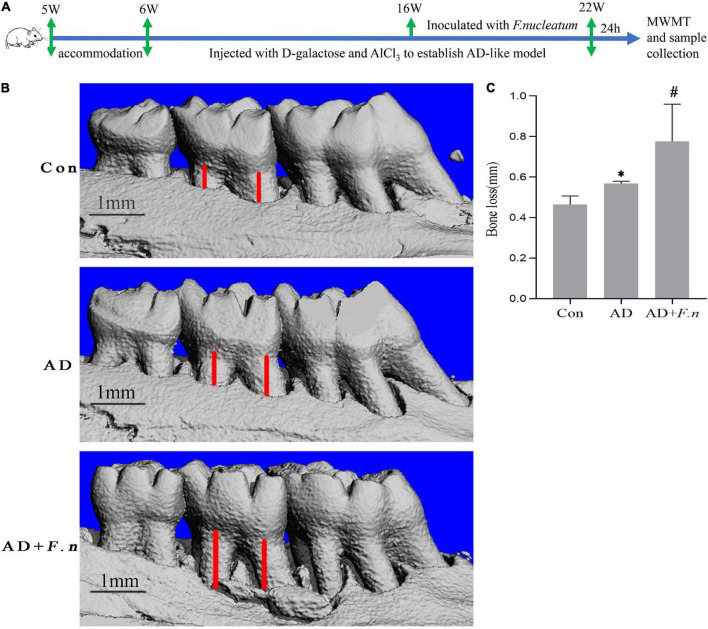
Micro-CT showed alveolar bone loss in each group (*n* = 4–5). **(A)** Flow chart. **(B)** Three-dimensional view of bone resorption. **(C)** Alveolar bone loss in each group. Data presented as mean ± SEM. *P < 0.05 vs. Con group, #*P* < 0.05 vs. AD group, indicates statistically significant differences.

After 1 week of environment acclimation, the rats were randomly divided into three groups, AD + ligation + non (AD, *n* = 4), AD + ligation + *F. nucleatum* (AD + *F.n*, *n* = 5), and blank (Con, *n* = 4). The rats were subcutaneously injected in the neck and back with D-galactose and AlCl_3_ (120 mg/kg/day and 10 mg/kg/day, 0.1–0.5 ml) or PBS every day from the 6th week to the 22nd week to establish an AD-like model (as shown in Figure 1A). Rats were ligated with a 5–0 silk suture around the bilateral maxillary second molars (M2) for experimental periodontitis in the 16th week. *F. nucleatum* ATCC 25586 was anaerobically grown and resuspended to a concentration of 1 × 10^9^ CFU/mL in 4% carboxymethyl cellulose (CMC), and was smeared on the silk suture (0.2 ml) every other day from the 16th week to the 22nd week (Chukkapalli et al., 2014). The Con group received 0.2 ml of PBS in 4% CMC.

### Morris water maze test

The Morris water maze test (MWMT, MT-200, Chengdu Taimeng Technology Co., Ltd.) was performed in the 23rd week to study the spatial learning and memory abilities of rats. Morris water maze test (MWMT) was carried out as described by Bhuvanendran et al. (2019) with few modifications. The water pool was a circular white pool with a diameter of 200 cm and a depth of 60 cm filled with non-toxic white paint opaque water and the temperature was held constant at 23 ± 1°C. The pool was separated into four equal quadrants, and an invisible platform was submerged 1 cm under the water surface in the center of the first quadrant and was considered the target quadrant. During the training days, the location of the platform remained the same and the rats were given tests for successive 4 days. In each trial, a rat was released in a quadrant and allowed to swim freely for 90 s to find the invisible platform and stay on it for 15 s. If the rats could not find the located platform in 90 s, it was gently guided to the platform, and allowed to stay there for 15 s. The time taken to reach the platform (escape latency) was recorded and the practice was repeated for four different starting quadrants of the trial per day. A probe trial test was performed on the 5th day without the platform to evaluate the memory consolidation rate. The rats were allowed to swim freely for 90 s, and count the number of times the mouse crossed the area where the platform had been placed.

### Specimen collection

Animals were sacrificed one day after the probe trial test. First, fresh feces of rats were collected for quick freezing. The rats were deeply anesthetized with an intraperitoneal injection of 2.5% tribromoethanol. After the collection of blood, the rats were sacrificed and the brain was collected. One cerebral hemisphere was immediately stored at −80°C until further analysis and the other half fixed in 4% paraformaldehyde for 24 h, then were processed and embedded in paraffin. Bilateral maxillary bones were removed and fixed in 4% paraformaldehyde for 24 h.

### Micro-computed tomography

To evaluate morphological changes in the alveolar bone, the maxillary bone was scanned by Micro-CT (Jia et al., 2019). Fixed maxillary bones of each group were randomly selected and scanned with a Micro-CT (μCT50; SCANCO) at the voxel resolution of 10 μm. Three-dimensional reconstruction and data analysis were performed by Scanco Evaluation. To calculate bone loss, the distance between the cementoenamel junction and alveolar bone crest (CEJ-ABC distance) was measured for two predetermined maxillary sites on palatal sides of M2.

### Hematoxylin-eosin staining

The histological changes in the sections of brain tissues from the different groups were observed using HE staining (Chen S. et al., 2017). After being preserved in 10% formalin solution for 24 h, brains were embedded in paraffin and 5 μm sections were sliced. The samples were then dewaxed and rehydrated, subsequently stained with hematoxylin and eosin, and examined under standard light microscopy for a general histopathology examination. The images were captured by a camera (Nikon, 90i, Tokyo, Japan).

### Immunohistochemistry

The immunohistochemical staining was performed as described by Shin et al. (2019) with slight modifications (Shin et al., 2019). After being fixed in 4% paraformaldehyde for 24 h, the brains were embedded in paraffin and then cut at a thickness of 5 μm. The sections for staining were deparaffinized and washed. Following, they were heated in 0.01 M sodium citrate buffer (pH 6.0) for antigen retrieval. Endogenous peroxide activity was quenched with 3% hydrogen peroxide. Sections were incubated with primary antibodies against Aβ1–42 (Abcam, ab10148, 1:200) overnight at 4°C, followed by anti-rabbit IgG (ZSGB-BIO, PV-9001). Then sections were incubated with horseradish enzyme labeled streptomycin working solution at room temperature for 30 min. Finally, color was developed with 3,3′-diaminobenzidine (ZSGB-BIO, ZLI-9018), and then it was counterstained with hematoxylin. After gradient dehydration with alcohol, the slices were sealed with neutral resin. The images were captured. And 6 visual fields per slices were being counted in a blind manner.

### Western blotting

The supernatant of brain protein was mixed with sample buffer and boiled. Protein from boiled samples was separated by 10% sodium dodecyl sulfate (SDS)-polyacrylamide gel electrophoresis and transferred to PVDF membranes. Then, the membranes were blocked in PBS–0.1% Tween 20 containing 5% milk and incubated with rabbit monoclonal IgG antibodies against GAPDH (1:1,000, Signalway Antibody, United States), Tau (phosphoThr181, 1:500, Signalway Antibody, United States), and mouse monoclonal IgG antibodies Tau (Tau46, 1:500, CST, United States) overnight at 4°C. The membranes were then incubated with goat anti-rabbit or rabbit anti-mouse IgG antibodies (1:5,000, Signalway Antibody, United States). The protein was visualized with Enhanced ECL Reagent Kit (Beijing Bio Excellence Biotechnology Co., Ltd., China). Images were captured by using a gel imaging system (Bio-Rad, United States).

### Limulus amebocyte lysate assay

Serum was isolated by centrifuging the blood after clotting at 4,000 rpm for 10 min. The serum level of LPS was measured with Tachypleus Amebocyte Lysate (EC64405, Xiamen Limulus Reagent Biotechnology Co., Ltd., xiamen, China) according to the manufacturer’s protocol.

### Brain genomic DNA isolation and polymerase chain reaction

To confirm the spread of periodontal pathogens from the mouth to the brain of AD-like rats, genomic DNA was isolated from the brains of all groups following the manufacturer’s protocol (DNeasy Blood and Tissue Kit, Qiagen, Germany) (Wang et al., 2015). DNA amplification was performed using a PCR amplification kit (Sangon Biotech Co., Ltd., Shanghai, China) according to the manufacturer’s instructions. Briefly, the PCR mixture contained 12.5 μl Taq PCR Master, 0.5 μl (10 μg/ml) DNA samples, 1 μl forward primer, 1 μl reverse primer, and 1 μl sterilized ddH2O. The primers used for amplification were as follows: 5′-GGCCACAAGGGGACTGAGACA-3′ (forward) and 5′-TTTAGCCGTCACTTCTTCTGTTGG-3′ (reverse) (Sangon Biotech Co., Ltd., Shanghai, China) and its reliability is verified. The reaction temperature was 94°C for 4 min, followed by 40 cycles of 94°C for 30 s, 60°C for 30 s, 72°C for 10 s, and then 72°C for 10 min. DNA amplification products were electrophoresed in a 2.0% agarose gel electrophoresis under 120 V for 20 min with *F. nucleatum* ATCC 25586 DNA as the positive group (183 bp) and a blank reaction system as the negative group.

### Fecal DNA extraction and 16S rRNA gene sequencing

DNA from 13 fecal samples of Con, AD, and AD + *F.n* groups were extracted using a Qiagen stool DNA extraction kit (Qiagen, Germany) according to the instructions. Eleven DNA samples (3 in Con, 3 in AD, 5 in AD + *F.n*), with ratios of 1.8–2.0 (for A260/280 nm) and > 1.8 (for A260/A230 nm), were used for downstream experiments (Wang et al., 2016). For the analysis of the composition of the gut microbiota, the V3 and V4 regions of the 16S rRNA gene were amplified using universal primers 338F and 806R. The sequencing was performed on the Illumina Miseq platform following the usual operating procedures of Personal Biotechnology Co., Ltd. (Shanghai, China). The DNA fragments were paired-end sequenced with the Illumina platform. The obtained sequences were denoise, quality controlled, dereplication, etc. to obtain high-quality sequences. The cluster_size module was used to cluster high quality sequences at a 97% similarity level, and the Operational taxonomic unit amplicon sequence variants (ASVs) tables were obtained. Finally, the singletons ASVs (ASVs detected in only 1 sample) and their representative sequences were removed from the ASV table. Calculated the length distribution of the high-quality sequences contained in the sample, and the obtained sequences were annotated with the Greengenes database. α diversity indices were determined using the Simpson index for diversity and the Chao1 index for species richness. β diversity can be demonstrated by Principal coordinate analysis (PCoA) and the Bray-Curtis distance was used to denote the β diversity distance (Li et al., 2019).

### Statistical analysis

All data were statistically analyzed by GraphPad Prism 6.0. Data were expressed as mean ± SEM. Morris’s water maze was analyzed with repeated measurements ANOVA. Comparisons between the three groups were evaluated by ANOVA or Kruskal-Wallis Test. Differences with *P* < 0.05 were considered statistically significant.

## Results

### *Fusobacterium nucleatum* increased alveolar bone loss

Micro-CT showed that the AD and AD + *F.n* groups had significantly more alveolar bone loss than the Con group, indicating the successful induction of periodontitis. Furthermore, alveolar bone loss in the AD + *F.n* group in rats infected with *F. nucleatum* for 6 weeks was increased compared with those of rats in the AD group (Figures 1B,C). These results suggested that oral infection with *F. nucleatum* induced more alveolar bone loss in AD-like periodontitis rats.

### *Fusobacterium nucleatum* damaged the cognitive ability of rats in Morris water maze test

**FIGURE 2:**
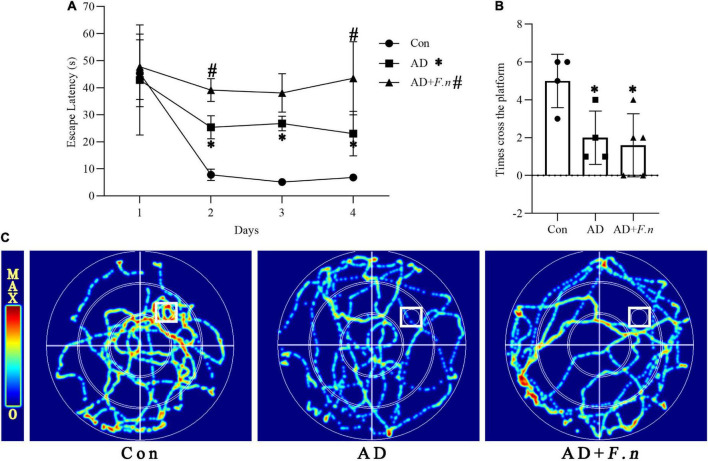
Morris water maze test (MWMT) showed the cognitive ability of rats in each group. **(A)** Escape latency. **(B)** The number of times cross the platform. **(C)** Heat map of rat movement track. Data presented as mean ± SEM. *n* = 4–5 in each group, *P < 0.05 vs. Con group, #*P* < 0.05 vs. AD group, indicates statistically significant differences.

To clarify whether *F. nucleatum* could induce cognitive impairment, we tested the learning ability of rats by MWMT. As shown in Figure 2A, the escape latency of the AD and AD + *F.n* groups were significantly increased from day 2 to day 4 when compared with the Con group (*P* < 0.05), indicating successful induction of the AD-like rat model through D-galactose and AlCl_3_ injection. In addition, when compared with the AD group, the escape latency of the AD + *F.n* group was significantly increased, in particular, there was an average increase of about 20 s on the fourth day (*P* < 0.05). These data indicated that chronic *F. nucleatum* oral infection could damage the learning ability further of AD-like with periodontitis rats.

In the space exploration experiment, we found that the track of rats in the Con group was mainly concentrated in and around the target quadrant (the first quadrant), while rats in the AD and AD + *F.n* groups moved disorderly or presented marginal movement, and rarely enter the target quadrant (Figure 2C). Moreover, the AD and AD + *F.n* groups had a significantly decreased number of times cross the platform than the Con group. These data also indicate the successful construction of the AD-like rat model. However, we didn’t find any evidence that *F. nucleatum* aggravated memory ability in rats as the AD + *F.n* group had a similar number of times cross the platform with the AD group (Figure 2B).

### *Fusobacterium nucleatum* could induce the expression of Aβ1–42 and p-Tau181

**FIGURE 3:**
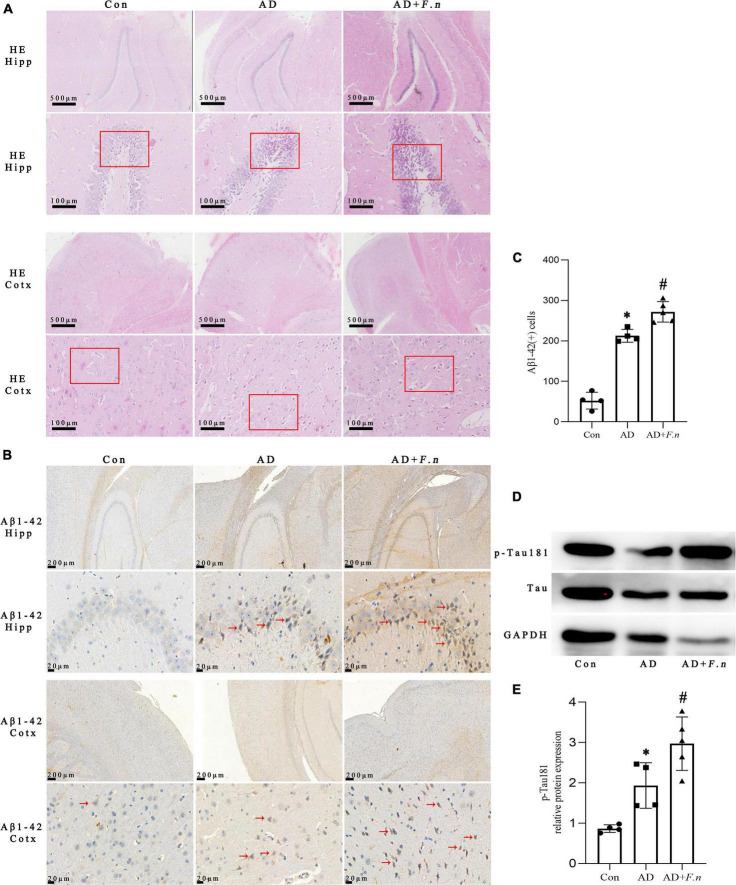
HE and IHC showed histopathological changes, and WB showed tau hyperphosphorylation in each group. **(A)** HE showed cell morphology and arrangement. **(B)** IHC showed the expression of Aβ1–42 cells. **(C)** The number of Aβ1–42 cells in the brain. **(D)** WB showed the expression of p-Tau181 in the brain. **(E)** Quantitative analysis of the blots. Data presented as mean ± SEM. *n* = 4–5 in each group, *P < 0.05 vs. Con group, #*P* < 0.05 vs. AD group, indicates statistically significant differences.

To clarify whether *F. nucleatum* could induce neurodegeneration, the neuronal morphology in the cortex (Cotx) and hippocampus (Hipp) of rats by HE were first investigated. We found that neurons in Cotx and Hipp of rats in the Con group were neatly arranged and compact, without significant cell vacuolation and necrosis (Figure 3A). Neuronal degeneration and karyopyknosis in brain tissue were observed in AD and AD + *F.n* groups, and cells in the hippocampus were loosely organized and vacuolation.

The Aβ1–42 expression in Cotx and Hipp of rats was examined by IHC. And the number of positive Aβ1–42 cells in brain tissue of AD and AD + *F.n* groups significantly increased (Figures 3B,C, *P* < 0.05). Compared with the AD group, the expression of Aβ1–42 in the AD + *F.n* group was significantly increased by about one-fourth (*P* < 0.05). These data indicated that chronic *F. nucleatum* oral infection could trigger neurodegeneration and Aβ1–42 express in the Cotx and Hipp of AD-like rats.

The expression of p-Tau181 and Tau protein in the brain of rats were detected by WB, as shown in Figures 3D,E. Compared with the Con group, the level of p-Tau181 in the AD group and AD + *F.n* group was significantly increased (*P* < 0.05). In addition, when compared with the AD group, the relative expression of p-Tau181 in AD + *F.n* group was significantly increased by about one-third (*P* < 0.05). These data indicated oral infection with *F. nucleatum* could induce phosphorylated tau increased in the brain of AD-like periodontitis rats.

### No *Fusobacterium nucleatum* was detected in the brain of rats following oral application of *Fusobacterium nucleatum*

We next explored the possible mechanisms by which *F. nucleatum*-caused cognitive impairment, neurodegeneration, and Aβ1–42 expression. PCR was used to examine whether *F. nucleatum* entered the brain first. None of the brain tissue lysates demonstrated DNA from *F. nucleatum* in the non-infected and infected groups (data not shown).

### Serum LPS increased after *Fusobacterium nucleatum* infection

**FIGURE 4:**
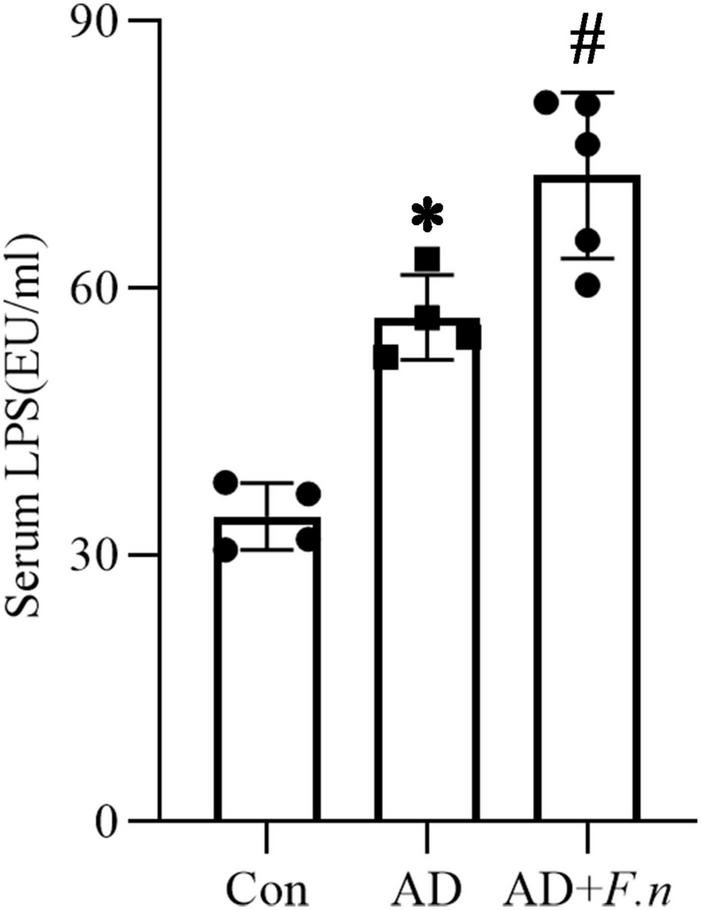
Serum LPS. Limulus amebocyte lysate (LAL) assay detected the serum LPS in three groups, *P < 0.05 vs. Con group, #*P* < 0.05 vs. AD group, indicates statistically significant differences.

Serum LPS of rats in each group was detected by LAL assay (as shown in Figure 4). Compared with the Con group, serum LPS of the AD group was significantly increased, suggesting the AD-like model could induce an increase in serum LPS levels, which may be related to systemic inflammation. Moreover, the AD + *F.n* group also had a significantly increased LPS level when compared with the AD group. These data suggested that *F. nucleatum* oral infection could trigger chronic systemic inflammation, we inferred that *F. nucleatum* might trigger neuropathy by inducing the release of LPS and the resulting inflammatory response in the circulatory system.

### *Fusobacterium nucleatum* could change the gut microbiota diversity of Alzheimer’s disease-like rats

A total of 866,530 high-quality sequences were captured from 11 fecal samples, with an average of 78,775 sequences per sample, and average length of 426 bp. The gut microflora of all samples was classified into 29 phyla, 89 classes, 158 orders, 280 families, 470 genera, and 5,763 ASVs.

**FIGURE 5:**
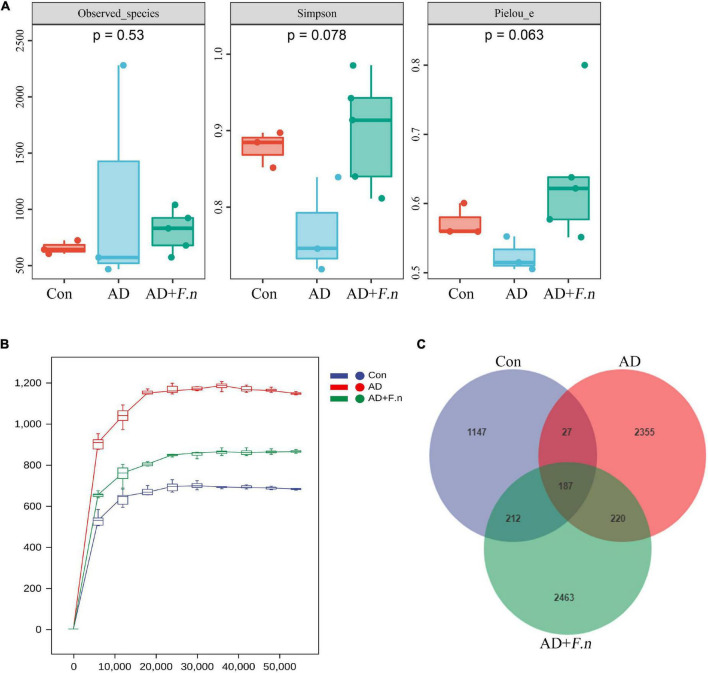
Gut microbiota diversity. **(A)** α diversity. **(B)** Rarefaction curves. **(C)** Venn diagram showing the unique shared ASVs in three groups.

α-diversity index of gut microflora was evaluated according to the observed species, Simpson, and Chao1 indices. As shown in Figure 5A, there was no significant difference in the three parameters among the three groups (*P* > 0.05). Regarding gut microflora structure, the dissimilarity tests showed that it was different between the AD group and the Con group to some extent (*P* < 0.1), but the AD group was similar to the AD + *F.n* group (*P* > 0.05; as shown in Table 1). The rarefaction analysis revealed that the sequence depth was almost sufficient to recover the diversity of this community (Figure 5B). Venn figure showed that there were, respectively, 1,573, 2,789, and 3,082 ASVs in Con, AD, and AD + *F.n* groups. Among them, AD and AD + *F.n* overlapped the most ASVs, 407. Only 187 ASVs were shared by three groups (Figure 5C).

**TABLE 1:** Comparison of the overall microbial community structure using ANOSIM.

Group 1	Group 2	*P*
Con	AD	0.087
AD	AD + *F.n*	0.463

### *Fusobacterium nucleatum* could change the gut microbiota composition and metabolism of Alzheimer’s disease-like rats

**FIGURE 6:**
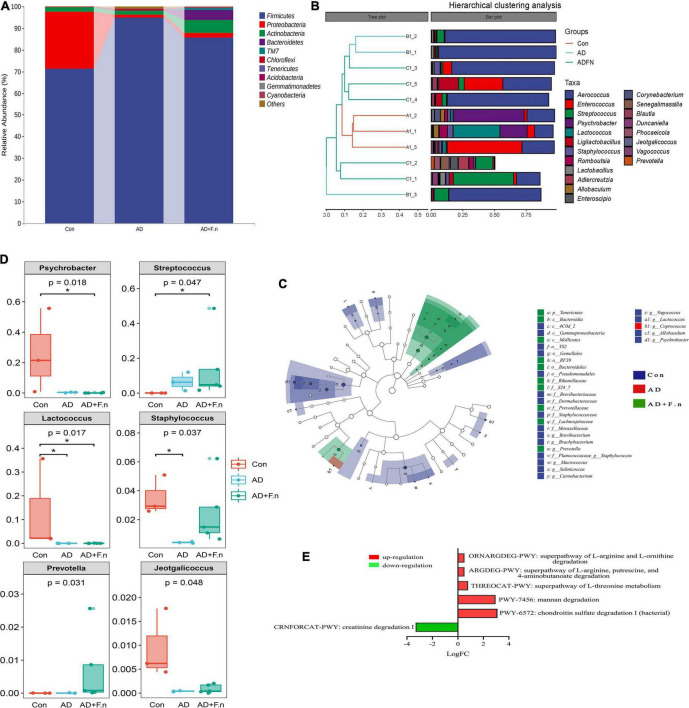
Gut microbiota composition and metabolism. **(A)** Classification and abundance of fecal bacteria at the phylum level (top 10) in each group. **(B)** Taxonomic classification and hierarchical clustering analysis of the bacterial communities at the genus levels from three groups (top 20). **(C)** The enriched taxa in the feces of rats were displayed in cladograms. **(D)** Significant difference in relative abundances of top 20 bacteria at the genus level in three groups. **(E)** Predicting metabolic pathways in MetaCyc, *P < 0.05.

At the level of phylum (Figure 6A), the top five phylum were *Firmicutes*, *Proteobacteria*, *Actinobacteria*, *Bacteroidetes*, and *TM7*. The relative abundance in the top five phylum had no significant differences between groups. According to hierarchical clustering analysis, the samples from the Con group (A1–3) clustered together and were well separated from the AD group (B1–3). Moreover, the AD + *F.n* group (C1–5) had a relatively nearer distance to AD (B1–3) compared to Con, which meant their community was more similar to each other. Although some genera were common to all samples, such as *Aerococcus*, the variability in genera distribution among different samples was noticeable (Figure 6B). Then, the structure and predominant taxa from the phylum to genus level of the gut microbial community in each group was evaluated by the linear discriminant analysis effect size (LEfSe) test and displayed in Figure 6C. Marked taxa from AD was *g_Coprococcus*, and most specific taxa were from AD + *F.n* group in phylum and genus were *p_Tenericutes* and *g_Prevotella*.

At the genus level, *Psychrobacter*, *Enterococcus*, and *Aerococcus* accounted for 50% of the community in the Con group, while in the AD group *Aerococcus* constituted 81.98%, AD + *F.n* group consists of the genera of *Aerococcus* and *Streptococcus* for 50% of the community. For the top 20 genera (Figure 6D), a variance analysis was performed and found that *Psychrobacter*, *Streptococcus*, *Lactococcus*, *Staphylococcus*, *Jeotgalicoccus*, and *Prevotella* have a differential expression. When compared with the AD group, the relative abundance of *Streptococcus* and *Prevotella* in the AD + *F.n* group was significantly increased.

PICRUSt2 was used to predict metabolic pathway in MetaCyc and found that some pathways were significantly increased or decreased in the AD + *F.n* group compared to the AD group (Figure 6E). Furthermore, pathways related to neurotransmitter degradation were significantly changed in the gut microbiome of *F. nucleatum*-treated rats compared with AD. ORNARGDEG-PWY, ARGDEG-PWY, THREOCAT-PWY, PWY-7456, and PWY-6572 involved in the degradation of L-arginine, L-ornithine, putrescine, 4-aminobutanoate, L-threonine, mannan, and chondroitin sulfate were significantly increased, while CRNFORCAT-PWY involved in the creatinine degradation decreased.

## Discussion

Periodontitis can cause inflammatory mediators to enter the blood circulation and further aggravate the development of some systemic diseases (Loos and Van Dyke, 2020). It is widely believed that Aβ deposition and phosphorylated tau may be associated with the local and surrounding inflammatory environment, especially when stimulated by gram-negative bacteria, which can accelerate Aβ accumulation and over-phosphorylated tau expression (Dioguardi et al., 2020; Tetz et al., 2020). This study has shown that periodontitis and its causative bacteria may also be associated with AD. Among them, the most studied is the relationship between *P. gingivalis* infection and AD. In this study, we demonstrated that periodontitis, caused by *F. nucleatum* infection, could exacerbate the pathological features of AD in an AD-like rat model.

D-galactose (D-gal) injected rodent models can recapitulate many features of AD, including cognitive deficits, neuronal degeneration, and apoptosis, and have been extensively applied in study (Chiroma et al., 2018). Aluminum chloride (AlCl_3_) can damage the membrane structure, induce neuronal fiber degeneration, inhibit nerve conduction, and lead to the unbalanced activity of α and β secretases (Sumathi et al., 2015). Treating rodents with D-gal + AlCl_3_ can result in impaired cognitive function and successfully build an AD-like model (Liaquat et al., 2017). In this experiment, rats after D-gal + AlCl_3_ treatment showed impaired spatial learning and memory ability, indicating that we successfully established an AD-like disease model in rats. It has been well established that *P. gingivalis* has a negative effect on cognitive function in animals with AD. For example, one study conducted behavioral tests and demonstrated that elderly C57BL/6J mice infected with *P. gingivalis* showed significant spatial learning and memory impairment compared with uninfected control mice (Ding et al., 2018). Furthermore, oral administration of *P. gingivalis*-LPS also can cause learning and memory impairments in C57BL/6 mice (Zhang et al., 2018). This study not only verified the established connection between *F. nucleatum* and AD but also found that oral infection of *F. nucleatum* can aggravate the learning disability of AD-like rats.

Periodontal infections serve as a significant risk factor affecting cognitive ability as demonstrated in a prospective observational clinical study in which cognitive decline is reported in AD patients with active chronic periodontitis compared to AD patients without active chronic periodontitis (Chen C.K. et al., 2017). Our results showed that oral infection with *F. nucleatum* can significantly increase the accumulation of Aβ and phosphorylated tau181 expression. Similarly, oral infection of wild-type mice with *P. gingivalis* for 22 weeks also resulted in a significant increase formation of Aβ, and the expression of p-Tau396 (Ilievski et al., 2018). It is reported that the LPS of gram-negative bacteria can act on the human immune system, inhibit the body’s immune defense, and then trigger inflammation (Morris et al., 2014). *In vivo* studies have also demonstrated that bacterial components, such as LPS and bacterial DNA, can lead to increased accumulation of Aβ and phosphorylated tau (Tetz et al., 2020). Some researchers conducted oral infection of *P. gingivalis* in 8-week-old wild C57BL/6 mice for 22 weeks and found that the Aβ plaque and surrounding LPS in the brain were significantly increased (Ilievski et al., 2018). In addition, LPS can co-locate with Aβ around the cerebrovascular area in patients with AD (Zhao et al., 2017). Regarding the ectopic detection rate of periodontal bacteria, different studies have different results. Animal experiments showed that orally infected with *P. gingivalis* for 3 weeks through pulp in wild type, and *P. gingivalis* was not detected in the brain (Foschi et al., 2006). Poole et al. showed that no bacterial DNA was found in the brain tissues after 12 and 24 weeks of oral infection of *Treponema denticola*, a periodontal pathogen (Poole et al., 2015). The present study did not detect *F. nucleatum* in the brain of rats, and the differences in these results may be related to the type of bacteria, the mouse model, and the time and route of infection.

In this study of gut microbiota diversity, we have found no significant difference in α- and β-diversity after D-gal + AlCl_3_ or *F. nucleatum* treatment. Bauerl et al. (2018) analyzed the gut microbiota of APP/PS1 transgenic AD mice and found that the α diversity of APP/PS1 mice aged 24 months and 6 months was lower than that of wild-type mice of the same age, and the gut microbiota structure of the two groups was significantly different. However, Bonfili et al. (2017) and Peng et al. (2018) found that there was no significant difference in α diversity between aging mice and 3 × Tg-AD mice and control mice, while β diversity showed a significant difference in gut microflora structure compared with control mice. The reason why our study is inconsistent with the above results may be associated with the animal species, AD model, and age.

Then, we found that after oral infection with *F. nucleatum*, the abundance of *Streptococcus* and *Prevotella* in the gut tract of AD-like rats was significantly increased. Clinical studies found that the abundance of *Streptococcus* is increased in the gut tract of patients with Parkinson’s disease, which can produce neurotoxins, such as streptomycin, streptomycin, and streptomycin, leading to permanent nerve damage (Li et al., 2017). In addition, *Streptococcus* is enriched in the gut microbiota of patients with colorectal cancer and has been associated with an increased risk of diseases, such as sepsis and endocarditis (Spigelblatt et al., 1985; Wang et al., 2012; Sutej et al., 2020). As for *Prevotella*, it is reported that the gut microbiota of patients newly diagnosed with AD was dysregulated, in which the abundance of *Prevotella*, which can promote inflammation, was significantly increased (Guo et al., 2021). In animal experiments, 16S rRNA gene sequencing analysis of gut microbiota of AD mice with accelerated aging also showed that the abundance of *Prevotella* was significantly increased (Peng et al., 2018). Among other neurological diseases, the abundance of *Prevotella* has been reported to correlate with the severity of Parkinson’s disease (Scheperjans et al., 2015). In addition, the successful colonization of *Prevotella* in the gut tract of mice can induce the production and accumulation of Th17 cells in the colon, and also increase the expression of Th17-related cytokines (interleukin 6/1β) in serum (Huang et al., 2020). Moreover, we found that *Lactococcus* and *Jeotgalicoccus* have a decreased relative abundance in the AD group. *Lactococcus* can convert glutamate into gamma-aminobutyric acid, a major inhibitory neurotransmitter, and abnormalities in this signaling pathway are associated with cognitive disorders, including AD (Gonzalez-Burgos et al., 2011). Moreover, another study reported that *Lactococcus lactis* can use as a treatment to restore the disturbed intestinal microbiota of Parkinson’s mice to the normal level, thus reducing neuroinflammation (Fang et al., 2020). *Jeotgalicoccus* can promote the fermentation of resistant starch and cellulose in the colon, and produce short-chain fatty acids (such as acetic acid, propionic acid, and butyric acid), in which butyric acid could reduce inflammation by reducing LPS translocation and inhibits the growth of facultative anaerobic bacteria to maintain the health of gut (Ege, 2017; Yang et al., 2017).

Next, we determined the predicted function of gut microbiota using the PICRUSt software, and we found through a MetaCyc pathway analysis that some enriched metabolites in *F. nucleatum* infection AD-like rats are related to amino acid and their derivatives metabolism (L-arginine degradation, L-threonine metabolism, L-ornithine, putrescine, 4-aminobutanoate degradation, and creatinine degradation) and carbohydrate metabolism (chondroitin sulfate degradation and mannan degradation) pathways. Studies have shown that changes in serum amino acid metabolism are associated with some systemic metabolic diseases (Newgard et al., 2009), for example, branched-chain amino acids contribute to the development of obesity-related insulin resistance, and aromatic amino acids (such as phenylalanine, tryptophan, and tyrosine) have also been confirmed to be involved in the pathogenesis of diabetes and cardiovascular diseases (Wang et al., 2011; Magnusson et al., 2013).

In this study, we chose co-injection of D-gal and AlCl_3_ to construct an animal model of AD, and found the chronic oral application of *F. nucleatum* can result in spatial learning impairment, neurodegeneration, Aβ formation with increased serum LPS, and increased abundance of *Streptococcus* and *Prevotella* in the gut tract. Whether this neuropathology is directly caused by serum LPS or gut microbiota is not clear and needs to be determined in future studies. The importance of our study is the demonstration that D-gal + AlCl_3_ induced AD-like rats can result in the accumulation of Aβ and phosphorylated tau181, increased serum LPS and abundance of *Streptococcus* and *Prevotella*, following chronic oral application of *F. nucleatum*.

In this study, using the AlCl3 + D-galactose induced AD-like rat model with periodontitis, we explored the possibility of *F. nucleatum* altering neurodegeneration and the Aβ1–42 formation in the brain. We also provide new evidence that the neuropathological features were greatly influenced by *F. nucleatum* infection and the consequential gut microbiota change. Our study strengthened the relationship between *F. nucleatum* and AD. The experimental basis supports the possibility of targeting microbial etiology for the treatment of AD.

## Data availability statement

The datasets presented in this study can be found in online repositories. The names of the repository/repositories and accession number(s) can be found below: https://ngdc.cncb.ac.cn/search/?dbId=&q=CRA004727.

## Ethics statement

The animal study was reviewed and approved by the Ethics Committees of the West China Hospital of Sichuan University (WCHSIRB-D-2021-009).

## Author contributions

CY wrote the manuscript and contributed to the data analysis. QD and YZ contributed to the manuscript writing. RH and YL conducted the study. CZ and XH contributed to data management and analysis. YL designed the study and analyzed the imaging data. All authors contributed to and approved the final manuscript.
